# Ruptured Ectopic Pregnancy in Caesarean Section Scar: A Case Report

**DOI:** 10.1155/2012/106892

**Published:** 2012-10-30

**Authors:** Kamal Singh, Anjali Soni, Shelly Rana

**Affiliations:** ^1^Department of Obstetrics and Gynecology, Dr. Rajendra Prasad Government Medical College Kangra at Tanda, Himachal Pradesh 176001, India; ^2^Department of Anesthesia, Dr. Rajendra Prasad Government Medical College Kangra at Tanda, Himachal Pradesh 176001, India

## Abstract

Pregnancy implantation within previous caesarean scar is one of the rarest locations for an ectopic pregnancy. Incidence of caesarean section is increasing worldwide and with more liberal use of transvaginal sonography, more cases of caesarean scar pregnancy are being diagnosed in early pregnancy thus allowing preservation of uterus and fertility. However, a delay in either diagnosis or treatment can lead to uterine rupture, hysterectomy, and significant maternal morbidity. We are reporting a rare case of first trimester caesarean scar pregnancy with viable fetus in the process of rupture, where uterine repair could be done, thus preserving the future fertility.

## 1. Introduction

Implantation of an ectopic pregnancy within a previous caesarean section scar is a rare condition. However, its incidence is increasing over the years due to the rise in caesarean section rates worldwide. A recent case series estimates an incidence of 1 : 2226 of all pregnancies, with a rate of 0.15% in women with a previous caesarean section and a rate of 6.1% of all ectopic pregnancies in women who had at least one caesarean delivery [1]. Caesarean scar pregnancy is potentially life threatening if not diagnosed and treated early. It may lead to catastrophic complications, such as uncontrolled haemorrhage and uterine rupture, which may require hysterectomy and results in subsequent loss of fertility. Majority of patients need immediate laparotomy after resuscitation but may need conservative approach if diagnosed early. Although expectant and medical managements have been reported, termination of a caesarean scar pregnancy by laparotomy and hysterotomy, with repair of the accompanying uterine scar dehiscence, may be the best treatment option [2].

## 2. The Case

**Figure 1:**
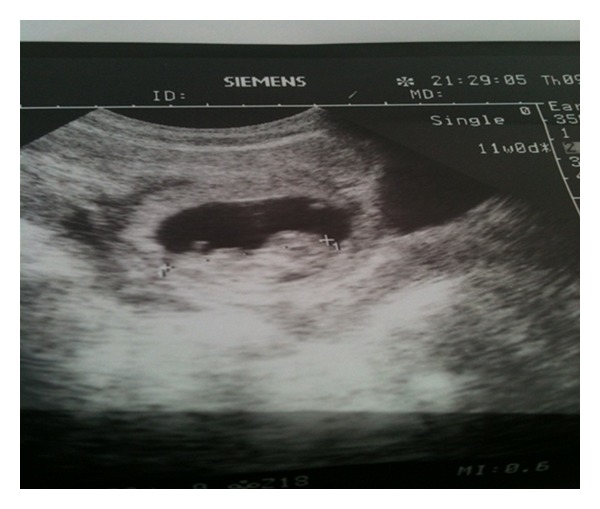
Transabdominal ultrasound showing gestational sac with fetus in the lower uterine segment.

A 24-year gravida 2, para 1, live 1 with confirmed pregnancy of 10-week 3-days gestation presented with acute abdomen. Her obstetric history was notable for one caesarean delivery two years back. In present pregnancy, she neither had any antenatal checkup nor any ultrasonography. On examination she had tachycardia and hypotension with moderate pallor. Abdomen was distended with evidence of free fluid and signs of peritonitis. Speculum examination revealed slight bleeding through cervical os. On bimanual examination uterus seemed enlarged; however, exact size could not be made out due to gross free fluid. She was resuscitated with fluids. Her haemoglobin was 5.5 gm%. Sonography showed an intact eccentrically located gestational sac with a viable fetus (CRL 11 weeks) in the anterior aspect of lower uterine segment scar (Figure 1) with free fluid in the peritoneal cavity.

**Figure 2:**
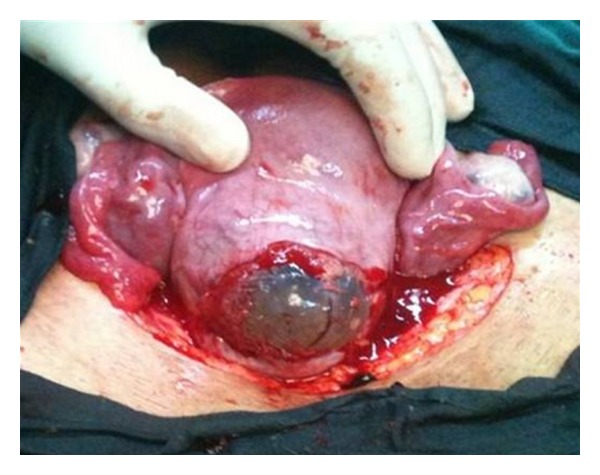
Intact gestational sac along with placental tissue seen protruding through previous caesarean scar defect.

Possibility of ruptured scar ectopic pregnancy was kept and exploratory laparotomy performed. Intraoperatively, we found one litre of haemoperitoneum with ruptured uterine scar through which amniotic sac was protruding (Figure 2). Uterus was evacuated and uterine defect repaired. Patient received two units of blood intraoperatively. Her postoperative period was uneventful and was discharged on the fifth postoperative day.

## 3. Discussion

Implantation of a gestational sac within a caesarean delivery scar is rarest form of ectopic pregnancy. A recent case series estimates an incidence of 1 : 2226 of all pregnancies, with a rate of 0.15% in women with a previous caesarean section and a rate of 6.1% of all ectopic pregnancies in women who had at least one caesarean delivery [1], although the overall prevalence seems to be increasing. There may be two subsets of caesarean scar pregnancies, first those that progress back toward the uterine cavity and may develop to term but with abnormal implantation and increased risk of bleeding, and those that progress towards the abdominal cavity with considerable risk of uterine rupture [3]. Sonography is the first-line diagnostic tool for scar pregnancy. A delay in diagnosis can lead to uterine rupture with a high risk of hysterectomy causing serious maternal morbidity and importantly loss of future fertility. There is also a danger of bladder invasion by the growing placenta. Today, serial serum hCG measurements and transvaginal ultrasound examination can provide early detection of most ectopic pregnancies. In those who require surgery, the type of procedure depends on the clinical situation and the location of the pregnancy [4].

The rarity of this entity results in a lack of consensus on optimal management. Reported strategies include expectant management, systemic methotrexate therapy, local injection of methotrexate, gestational sac aspiration, dilatation and curettage, surgical laparotomy/hysterotomy, hysteroscopy, laparoscopy, and uterine artery embolization [5, 6]. Haimov-Kochman et al. suggested that noninvasive therapy should be considered in suitable cases of caesarean scar ectopic pregnancy. In cases discovered at no more than 6–8 week's gestation without fetal cardiac activity, methotrexate injection and expectant management may be a safe treatment alternative [7]. Ultimately, the approach depends on various factors such as gestational age at presentation, hemodynamic stability, local endoscopic expertise, future fertility plans, and feasibility of serial follow-up serology and imaging.

## 4. Conclusion

Uterine rupture during first trimester of pregnancy is an extremely rare, but life-threatening cause of intraperitoneal haemorrhage. The ectopic pregnancy within the scar of a previous caesarean delivery is best diagnosed by transvaginal ultrasound. However, a delay in either diagnosis or treatment can lead to uterine rupture, hysterectomy, and significant maternal morbidity. Though a rare event, the incidence of caesarean scar pregnancy seems to be on the rise due to increasing caesarean section rate. Hence, an obstetrician is likely to encounter this entity in his or her lifetime. Heightened awareness amongst obstetricians regarding the possibility of scar pregnancy in those with prior caesarean section and early ultrasound in these women may lead to early diagnosis and hence a chance of conservative management.
